# Granulosa cell tumors and menopausal hormone therapy: A narrative review

**DOI:** 10.1016/j.gore.2026.102123

**Published:** 2026-06-01

**Authors:** Abigail Shore, Lanlan Fang, Shelley Hewins, David G. Huntsman, Lesa M. Dawson

**Affiliations:** a Department of Obstetrics and Gynaecology, Faculty of Medicine, University of British Columbia, 2194 Health Sciences Mall, Vancouver, BC V6T 1Z3, Canada; b Department of Pathology and Laboratory Medicine, University of British Columbia, 2194 Health Sciences Mall, Vancouver, BC V6T 1Z3, Canada; c Patient Partner, Canada; d BC Cancer Research Institute, 675 W 10th Ave, Vancouver, BC V5Z 0B4, Canada; e Division of Gynecologic Oncology, Department of Obstetrics and Gynaecology, University of British Columbia, 2775 Laurel Street, Vancouver, BC V5Z 1M9, Canada

**Keywords:** Granulosa Cell Tumor, Menopausal Hormone Therapy, Survivorship, Quality of life

## Abstract

Adult granulosa cell tumors (GCT) are rare sex cord stromal tumors of the ovary, characterized by distinct histologic features and the near ubiquitous presence of a recurrent somatic mutation in the FOXL2 gene. Although GCT have a favorable prognosis compared to other ovarian cancer subtypes, recurrences typically occur later than other ovarian cancers; usually between 4–8 years after initial diagnosis. Once recurrence is diagnosed, cure through surgery is rare and few effective systemic therapies exist. The median age at GCT presentation is between 46–50 years old, and many cases occur in premenopausal women. GCT are hormonally active tumors, with many of the tumors secreting estrogen. Hormone receptors, including progesterone and estrogen receptors are often expressed in these tumors.

Younger GCT patients who undergo the recommended surgical staging procedure, experience premature surgical menopause, which is associated with vasomotor symptoms, increased risk of osteoporosis and cardiovascular events as well as reduced cognitive and sexual functioning. Menopausal hormone therapy (MHT) mitigates these risks and is recommended for individuals with premature menopause. In GCT patients, most guidelines caution against MHT use, due to perceived risk associated with the hormonally and endocrinologically active nature of these tumors. This narrative review outlines the hormonal nature of GCT and summarizes and synthesizes the evidence relating to possible safety and risk of MHT use in GCT patients.

## Highlights

• Granulosa cell tumors are rare sex cord stromal tumors and have a favorable prognosis at primary presentation.
• Younger women with these tumors experience premature menopause, and menopausal hormone therapy is recommended treatment.
• Menopausal hormone therapy is not recommended for granulosa cell tumors because they are hormonally active.
• There is no evidence to suggest safety or risk of initiating menopausal hormone therapy in granulosa cell tumor patients.

## 1 Introduction

Adult Granulosa cell tumors (GCT) are the most common sex cord stromal tumors and constitute approximately 2–5% of all ovarian tumours. (Jamieson and Fuller, 2012) The median age at GCT presentation is between 46–50 years old, but many cases occur in younger women who are premenopausal. (Pilsworth, 2021, Sun, 2012, Plett, 2023, Levin et al., 2018) A case series of 176 granulosa cell tumor patients in Taiwan found that 32.9% of these patients were diagnosed in the fourth decade of life or younger. (Sun, 2012) Another study of 168 tumors found that 44.6% of GCT patients were < 46 years of age at diagnosis. (Plett, 2023) The discovery of the *FOXL2* point mutation, characteristic of 95% of granulosa cell tumors, was a landmark finding and greatly enhanced understanding of the pathogenesis and identification of these tumors. (Pilsworth, 2021).

GCT are associated with high initial survival rates as 50–90% of patients present with stage I disease, and prognosis is highly dependent on stage. (Jamieson and Fuller, 2012, Bryk, 2015) Time to recurrence for GCT is usually between 4–8 years, with the median time being 7.2 years. (Pilsworth, 2021, Sun, 2012) Recurrences tend to be associated with significant morbidity and mortality, with around 50–80% of those with recurrent tumors succumbing to their disease. (Jamieson and Fuller, 2012) Between 10–64% of GCT recur. (Sun, 2012, Levin et al., 2018, Bryk, 2015, Van Meurs, 2014) Initial treatment for stage I disease is typically bilateral salpingo-oophorectomy and staging, or unilateral oophorectomy in those desiring fertility preservation. (Jamieson and Fuller, 2012).

Premature menopause after bilateral oophorectomy is associated with increased risk of cardiovascular disease, bone loss and cognitive decline, as well as vasomotor symptoms and sexual distress. (Rees, 2020) Menopausal hormone therapy (MHT) can greatly reduce these risks. (Rees, 2020) Current recommendations caution against MHT use in GCT patients, based on concern about hormonal activity and receptors. (Rees, 2020, Crean-Tate et al., 2020, Cagnacci, 2025) GCT are hormonally active tumors, often secreting estradiol (E2), Inhibins and Anti Mullerian Hormone. (Pilsworth, 2021, Färkkilä, 2015, Chen et al., 2020) GCT also express various hormone receptors, including estrogen receptors (ER) and progesterone receptors (PR). (Němejcová, 2024, Liu, 2023, Moh, 2024) Hormonal treatments, such as aromatase inhibitors, have been used with some success to treat GCT. (Van Meurs et al., 2014, Banerjee, 2021).

The goal of this narrative review is to dissect the hormonal nature of GCT–this is the main reason MHT is not recommended for use in GCT patients–and summarize and synthesize the evidence relating to possible safety and risk of MHT use in GCT patients. We seek to consolidate what is known about the hormonal nature of GCT, identify gaps in the literature and inform future research.

## 2 Methods

For this review, potentially relevant articles were identified by a search in the Medline database and Web of Science database using both Medical Subject Headings (MeSH) terms and free text words searched in Title and Abstract: ((Granulosa Cell Tumour [MeSH Terms]) OR (Sex cord stromal tumour) AND ((estradiol) OR (hormone therapy) OR (estrogen) OR (estrogen replacement therapy) OR (progesterone receptors) OR (progesterone) OR (ERα) or (ERβ) OR (G coupled protein receptors) OR (hormone replacement therapy) OR (menopausal hormone therapy). We cross referenced publications found in this search to identify further relevant publications. We chose these search terms to try and capture any literature which relates to the hormonal nature of GCT, and to capture literature exploring MHT in relation to GCT. To capture the most recent literature a preference for papers released during and after 2010 was given. We excluded papers on juvenile GCT, on hormones not relevant to MHT (i.e. androgens), those focused on other types of tumors, those relating to non-hormonal GCT treatment, and non-English language papers. As this is a narrative review, we are not providing a systematic synthesis of the literature but instead are reporting a summary of the most relevant papers based on our judgement and the goals of this review.

## 3 Normal granulosa cell functioning

Granulosa cell tumors (GCT) arise from the granulosa cell layer of the ovary. (Birgersson, 2023) Granulosa cells, together with cumulus and theca cells, surround the oocyte in the ovarian follicle. (Birgersson, 2023) The major function of granulosa cells is to produce steroid hormones, cytokines, and growth factors that are required for ovarian physiology. (Field et al., 2014, Fang et al., 2023).

### 3.1 Estrogen synthesis and function

During the reproductive years, granulosa cells are the major location of estradiol (E2) production. (Birgersson, 2023) According to the two-cell, two-gonadotropin hypothesis, while luteinizing hormone (LH) induces androgen production in theca cells, follicle-stimulating hormone (FSH) stimulates aromatase activity in the granulosa cells and synthesizes estrogen. (Hillier et al., 1994) E2 is the predominant form of estrogen and is responsible for primary and secondary sexual characteristics as well as regulating the menstrual cycle and fertility. (Birgersson, 2023) Aromatase; a member of the cytochrome P450 superfamily, is a key enzyme in granulosa cells and is responsible for the last step in E2 synthesis. (Barbieri, 2024).

When granulosa cells proliferate in response to rising FSH, they begin to produce more E2. (Barbieri, 2024) Pituitary gonadotropin stimulation is required for follicles to undergo ovulation and coincides with increased FSH receptor presence, allowing enhanced follicle growth. (Barbieri, 2024) FSH binding triggers a signalling cascade, which culminates in the expression of a variety of target genes required for cellular proliferation and differentiation, allowing ovulation. (Barbieri, 2014, Channing et al., 1980) After ovulation, E2 and progesterone—which is produced by the luteinized granulosa cells—trigger negative feedback from the hypothalamus and anterior pituitary, maintaining low FSH and LH levels. (Barbieri, 2024).

### 3.2 Estrogen receptor signalling

**Fig. 1:**
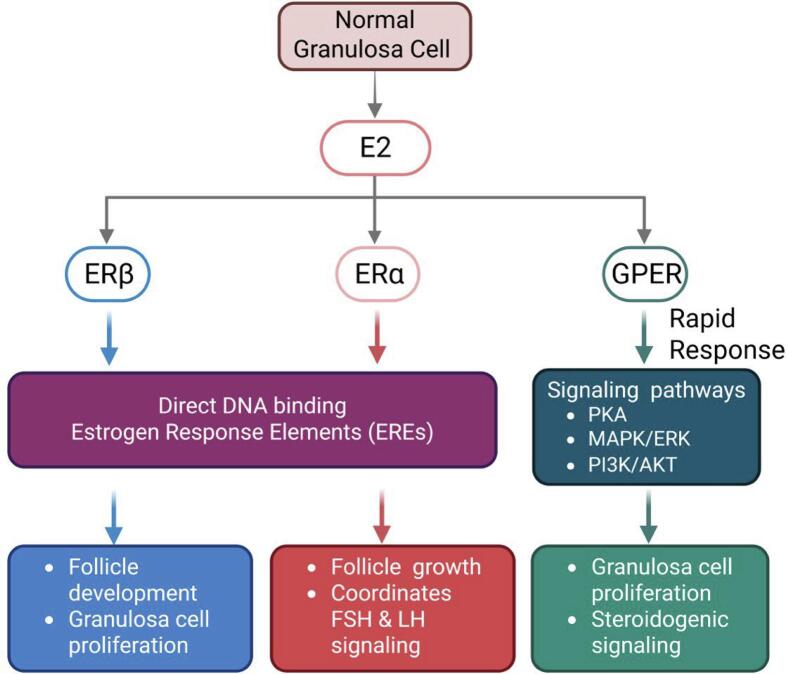
Summary of estrogen signaling in granulosa cells. E2 = Estradiol, GPER = G protein-coupled estrogen receptor, FSH = Follicle stimulating hormone, LH = luteinizing hormone.

E2 signals through two nuclear ERs, estrogen receptor alpha (ERα) and estrogen receptor beta (ERβ), or the membrane receptor G-coupled protein receptor 1 (GPER). (Chauvin et al., 2022)GPER has a relatively low affinity for estrogens. (Birgersson, 2023) In general, estrogen receptors (ER) and estrogen signalling vary drastically between tissues. (Deli et al., 2020) ERα is expressed in the uterus, ovaries, breast, and bone, whereas ERβ is predominantly expressed in granulosa cells. (Chauvin et al., 2022) There are various ERβ isoforms which are expressed at different levels throughout the menstrual cycle in granulosa cells. (Chauvin et al., 2022) ERα and ERβ exert their effects through E2 binding to estrogen response elements and this binding can be mediated by transcription factors. (Deli et al., 2020) Differential expression levels of each ERβ isoform may be important in E2 action and sensitivity, although this is not well understood. (Chauvin et al., 2022) Given the different receptors that E2 can signal through, the different ERβ isoforms, their different expression levels, and different responses based on E2 levels, the effect of E2 on its receptors is complex. Fig. 1 outlines estrogen signalling in normal granulosa cells.

### 3.3 Progesterone synthesis, functioning and signalling

After ovulation, LH stimulates granulosa cells to produce progesterone. (Barbieri, 2024) The elevated progesterone levels after the LH surge are involved in the regulation of follicular rupture and luteinization, which play critical roles in maintaining a successful pregnancy at the early embryonic stage. (Barbieri, 2024) If no implantation occurs, progesterone levels decrease and menstruation occurs. (Barbieri, 2024) Progesterone binds to nuclear progesterone receptors (PR) within the cytoplasm of the cell, where it will undergo dimerization and translocation to the nucleus to bind to DNA and regulate gene expression. (Puechl, 2019) PR receptors have 3 isoforms, PR-A, PR-B and PR-C. PR-A and PR-B are the predominant isoforms in granulosa cells. (Puechl, 2019).

### 3.4 FOXL2 in granulosa cells

*FOXL2* is an important gene in ovarian differentiation and is expressed in eyelids and in adult and fetal ovarian follicular cells. (Jamieson and Fuller, 2012) Wildtype *FOXL2* is necessary for developing and maintaining the ovarian phenotype and supports follicular growth through granulosa cell differentiation. (Yang et al., 2018) Furthermore, wildtype *FOXL2* has important roles in regulation of cell death, proliferation and tumorigenesis, as well as regulation of steroid hormone production. (Birgersson, 2023) CYP19A1, or aromatase is upregulated by *FOXL2* and in turn, E2 has been shown to regulate *FOXL2* expression in granulosa cells*.* (Yang et al., 2018, Färkkilä, 2017, Ito, 2022).

## 4 GCT Molecular characteristics

### 4.1 Reproductive hormones

GCTs secrete estradiol (E2), inhibin B, and anti-mullerian hormone (AMH). (Haltia, 2020) The abnormal bleeding that many GCTs present with is associated with high levels of E2 secretion and patients can present with estrogen-induced endometrial hyperplasia. (Bryk, 2015, Haltia, 2020) GCTs secrete increased levels of E2 due to high and unregulated aromatase expression. (Jamieson and Fuller, 2012) Despite the majority of GCT secreting high levels of E2, up to 30% may not produce the hormone. (Salkeni et al., 2024).

#### 4.1.1 Anti-mullerian hormone and inhibin B

GCTs produce Inhibin B, and there is correlation between inhibin B levels and tumor size. (Bryk, 2015, Färkkilä, 2017) Inhibins are glycoprotein hormones synthesized predominantly by the granulosa cells in response to FSH from the anterior pituitary. (François, 2015) In addition to functioning as growth factors, Inhibins also regulate the synthesis and secretion of FSH through a negative feedback loop. As such, high serum Inhibin B levels are associated with supressed FSH plasma levels in GCT patients, which indicates that the Inhibin B released by GCT is biologically active. (Jamieson and Fuller, 2012) Anti-mullerian Hormone (AMH) is also secreted by GCTs. (Bryk, 2015) This hormone is expressed by granulosa cells during the reproductive years and mediates the formation of primary follicles. (Jamieson and Fuller, 2012) Both serum Inhibin B and AMH were found to be significantly elevated in patients with primary and recurrent GCTs and the levels were found to be associated with tumor size. (Färkkilä, 2015) A 2020 *meta*-analysis of 5 papers found that AMH is an accurate serum biomarker in GCT diagnosis, with 89% pooled sensitivity, 93% pooled specificity. (Cluzet, 2022).

### 4.2 Hormone receptors

Multiple different hormonal receptors have been characterized on GCTs. A recent immunohistochemical analysis of 290 GCT cases found expression of estrogen receptor (ER) in 41%, progesterone receptor (PR) in 94%, and androgen receptor (AR) in 82% of cases. (Němejcová, 2024) This is consistent with previous studies, with ranges of 16–66%, 5–100%, and 59–100% reported for ER, PR and AR, respectively. (Němejcová, 2024) These large ranges likely represent the large amount of variability in expression levels between different GCTs. (Monteiro et al., 2024) Differing observations can also be attributed to small sample sizes in many studies..() There is also some concern that previous work did not use validated antibodies for receptor detection, and therefore some past research on hormone receptors on GCT may not be accurate. (Moh, 2024).

### 4.3 Estrogen signaling in GCT

**Fig. 2:**
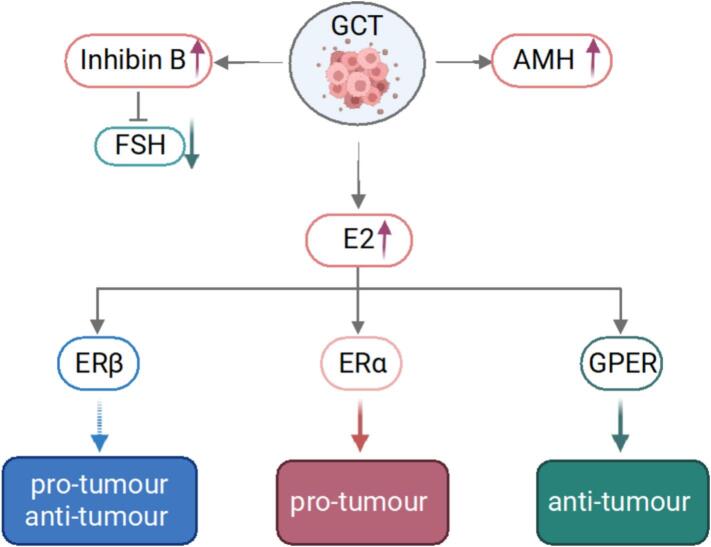
Summary of estrogen signaling in GCT. FSH = Follicle stimulating hormone, GCT = Granulosa cell tumor, AMH = Anti-mullerian hormone, E2 = Estradiol, GPER = G-coupled protein receptor 1.

Given the complexity of E2 signalling in granulosa cells, it has been difficult to understand the mechanisms of E2 signalling in GCT. (Chauvin et al., 2022) See Fig. 2 for a brief summary of E2 action in GCTs. In general, ERβ is thought to have an anti-proliferative effect while ERα is thought to induce proliferation. (Deli et al., 2020) In epithelial ovarian cancers, ERβ takes on a tumor suppressor role, wherein it opposes the pro-carcinogenic effects of ERα. (Ciucci, 2018) However, the role of ERβ in granulosa cells may be different, as this receptor has been proposed to promote cell proliferation and survival in granulosa cells during folliculogenesis. (Monteiro et al., 2024) This suggests that ERβ can have a pro- or anti-tumour role depending on the tissue in question. (Chauvin et al., 2022) This variation may be related to the presence of different ERβ isoforms, which may play different roles. (Chauvin et al., 2022) Multiple studies have indicated that activation of ERβ may have a tumour-promoting role in GCT. (Monteiro et al., 2024, Rosario et al., 2012) Cluzet et al. suggest a mechanism by which ERβ-mediated tumour cell growth is dependent on the presence of ERα, proposing that in the absence of both ERs, E2 could prevent metastatic spreading through GPER. (Monteiro et al., 2024) Ciucci et al. suggest an anti-apoptotic role of E2 when it acts on ERβ. (Rosario et al., 2012) Haltia et al. also present results which suggest that E2 may promote GCT growth, although only at very high E2 doses. (Haltia, 2020).

Francois et al. showed that E2 inhibited metastasis in GCT through action at the GPER receptor. (François, 2015) Cluzet et al. also found that GPER may inhibit metastatic spreading, and speculate that this effect is not shown when ERs are present, due to preferential E2 binding to ERs. (Monteiro et al., 2024) In summary, there is no conclusive understanding of the impact of E2 on GCT or of E2 signalling within GCT, although there is evidence to suggest that ERβ and ERα activation may have a proliferative effect, and that the effect of E2 on GCT is highly dependent on the receptors present.

### 4.4 Progesterone signalling in GCT

There is very little known about the role of progesterone signalling in GCT. One study by Puechl et al. suggested that higher PR presence was associated with decreased progression free survival and overall survival in GCT. (Puechl, 2019) PR-A and PR-B are thought to have overlapping transcriptional responses which result in proliferative and anti-proliferative action, depending on the tissue and context. (Puechl, 2019) Overall, the role of progesterone is GCT is not well understood or described.

### 4.5 FOXL2 in Granulosa Cell Tumours

Nearly all adult GCTs are characterized by a single somatic missense mutation (C134W) in the *FOXL2* gene. (Jamieson and Fuller, 2012) There is evidence that mutated *FOXL2* gains oncogenic function, and the mutated protein remains in the nucleus and is transcriptionally active. (Jamieson and Fuller, 2012, Birgersson, 2023) Furthermore, aromatase is a direct transcriptional target of mutated *FOXL2,* which leads to excessive estrogen levels in GCT patients. (Birgersson, 2023, Singh and Oehler, 2010).

## 5 Hormone treatments for GCTs

Given the hormonal activity of GCTs, multiple hormone treatments have been developed for these tumors. The hormonal treatment with the most evidence of efficacy is aromatase inhibitors. (Banerjee, 2021, Yang et al., 2018) The other types of hormonal treatments for GCT are GnRH agonists and antagonists, and hormone receptor acting agents such as megestrol acetate, medroxyprogesterone, tamoxifen and diethylstilbestrol. (Yang et al., 2018).

### 5.1 Aromatase Inhibitors

The main aromatase inhibitors which have been used to treat GCT are anastrozole and letrozole. They exhibit a strong anti-estrogen effect and may result in a decrease of up to 90% of the aromatization of androgens by aromatase. (Van Meurs et al., 2014) These treatments work to target the aromatase overexpression which characterizes many GCTs. A systematic review of all hormone treatments for GCT in 2014 found that users of aromatase inhibitors had the most complete and partial responses compared to other treatments. (Van Meurs et al., 2014) 5/5 patients on anastrozole had a partial or complete response and 4/4 patients on letrozole had a partial or complete response. (Van Meurs et al., 2014) However, the PARAGON study—a phase II single arm, open label, multicenter trial published in 2021—reported much more modest response rates. (Banerjee, 2021) In 41 patients with ER/PR positive GCT, 10.5% had an objective response to anastrozole, although 80% had clinical benefit at 12 weeks and 60% were progression free at six months. (Banerjee, 2021) Although these results indicate some benefit, the response is much more modest than previously reported.

### 5.2 Other Hormone Therapies

GnRH agonists desensitize, downregulate or block pituitary GnRH receptors, which inhibits the production of gonadotropins. (Van Meurs et al., 2014) They are often used in tandem with aromatase inhibitors, and have only shown modest efficacy. (Van Meurs et al., 2014) In 16 patients treated with a GnRH agonist, 31% had a response. In the same *meta*-analysis 1 patient was reported to have been treated with a GnRH antagonist, with a 0% response rate. (Yang et al., 2018) Progestins such as megestrol acetate and medroxyprogesterone acetate (MPA) reduce estrogen secretion by inhibiting the production of pituitary gonadotropins. A review of multiple hormone treatments suggested that as single agents, megestrol acetate had a response rate of 36% (4/11 patients) and MPA had a response rate of 100% (4/4 patients). (Yang et al., 2018) Tamoxifen is an antiestrogen which binds to ER competitively. It is generally used to block estrogen in breast cancers. (Van Meurs et al., 2014) As a monotherapy for GCT, tamoxifen has shown very little benefit. (Van Meurs et al., 2014) In 12 patients treated with tamoxifen, the response rate was 8%. (Yang et al., 2018).

In conclusion, while hormone therapy has shown some benefit in GCT, the findings are mixed and suggest heterogeneity in which tumors respond to treatment.

## 6 Menopausal hormone therapy

Menopausal hormone therapy (MHT) in the form of systemic estrogen-based therapy has a favorable risk–benefit profile in women without cancer who are younger than 60 or for 10 years after menopause. (Rees, 2020) Progestogens are added to regimens for women with intact uteruses to avoid endometrial hyperplasia and carcinoma. (Rees, 2020) In women with premature menopause, MHT is recommended until at least the age of natural menopause, to reduce the risks associated with premature menopause, including osteoporosis, cardiovascular risk and cognitive decline. (Rees, 2020) Additionally, MHT can greatly improve vasomotor symptoms, sleep disruption, mood, sexual functioning and overall quality of life. (Rees, 2020).

As described by Deli et al., the factors taken into consideration when making decisions about MHT for cancer survivors include:().

1. The oncologic characteristics of the tumor and previous and current treatments
2. Details of the planned MHT regimen
3. Endocrine characteristics of the previous tumor (i.e. type of hormones released and hormone receptors present)
4. Former or current hormone oncotherapy
5. The effect of progesterone and estrogen on the given tissue or specific tumor type, if known

Evidently, decisions around MHT can be complicated, especially in rarer tumors like GCT, where less research is available on the safety of administration.

### 6.1 MHT recommendations with GCT

Currently, the bulk of the literature recommends against using MHT in individuals who have had GCTs (Table 1). While there is no direct evidence supporting this recommendation, it is thought that it is a safer option not to initiate MHT in these patients, due to the hormonally and endocrinologically active character of these tumors. (Deli et al., 2020).

**Table 1:** Summary of recommendations for MHT use in those with previous GCT 2010-present.

Author(s)	Paper Type and/or Recommending Body	Recommendation	Citations
Singh and Oehler, 2010 (Rousset-Jablonski, 2019)	Review (unspecified)	• General belief is that MHT should not be used, as GCTs are endocrinologically and hormonally dependent	• None
Biliatis at al., 2012 (Guidozzi, 2013)	Review (unspecified)	• MHT should not be used for GCT, as they are considered hormone dependent tumours	• None
Guidozzi, 2013 (Kuhle, 2016)	Review (unspecified)	• May be prudent to avoid estrogen therapy in survivors of GCT	• None
Kuhle et al., 2016 (O’Donnell et al., 2016)	Narrative Review	• No data on the safety of MHT after GCT, but MHT is generally avoided as GCTs are hormonally active	• Singh and Oehler, 2010 (Rousset-Jablonski, 2019)
O’Donnell et al., 2016 (Del Carmen and Rice, 2017)	Review (unspecified)	• GCTs are hormonally dependent and MHT should be avoided after diagnosis	• Biliatis et al., 2012 (Guidozzi, 2013)
Del Carmen and Rice, 2017 (Ray-Coquard, 2018)	Review (unspecified)	• No studies to refute or support use of MHT • Recommend avoiding MHT after GCT are they are hormonally active and hormone dependent	• Singh and Oehler, 2010 (Rousset-Jablonski, 2019)
Ray-Coquard et al., 2018 (Hickey, 2024)	European Society for Medical Oncology Guidelines	• MHT should be avoided, as GCTs are thought to be hormone dependent	• None
Rousset-Jablonski et al., 2019	French National Network Dedicated to Rare Gynaecological Cancers guidelines	• MHT indication should be discussed on a case to case basis in a multidisciplinary team meeting	• None
Deli et al., 2020 (Deli et al., 2020)	Review (unspecified)	• No direct evidence to prove or disprove long-term negative effect of MHT on GCT survivors • May be safer to not initiate MHT due to the endocrinologically active character of these tumours	• Singh and Oehler, 2010 (Rousset-Jablonski, 2019) • Kuhle et al., 2016 (O’Donnell et al., 2016) • Del Carmen et al., 2017 (Ray-Coquard, 2018)
Rees et al, 2020 (Rees, 2020)	European Menopause and Andropause Society and International Gynecologic Cancer Society position statement	• Generally believed that estrogens should not be used, as GCTs are hormone dependent • No study has demonstrated a deleterious effect of MHT	• None
Crean-Tate et al., 2020 (Crean-Tate et al., 2020)	Expert Review	• GCTs are hormonally active and patients are generally advised to avoid systemic MHT	• Deli et al., 2020 (Deli et al., 2020)
Cagnacci et al., 2024 (Cagnacci, 2025)	Italian Group of Study for Management of the Menopause Consensus Opinion	• MHT is allowed after non-epithelial ovarian tumors with the exception of GCT	• None
Moss et al., 2024 (Boyce, 2009)	British Gynaecological Cancer Society Practice Recommendations	• No direct evidence to support or refute the long-term negative effect of MHT on granulosa cell tumour survivors, but considering the endocrinologically active character of these tumours, it may be safer not to initiate MHT in those with advanced disease	• Deli et al., 2020 (Deli et al., 2020)
Hickey et al., 2024^61^	Review	• European guidelines suggest avoiding MHT, might be oestrogen sensitive	• Rees et al., (2020) (Rees, 2020)
Taylor et al., 2024 (Moss, 2024)	British Gynaecological Cancer Society and British Menopause Society guidelines	• Limited evidence does not demonstrate harm with MHT following treatment for stage I GCT, but tumours are hormone-sensitive and women should be counselled regarding uncertainties • MHT use, especially in advanced disease, would depend on treatment regimens, individual symptoms and risk/benefit discussions	• Rees et al., (2020) (Rees, 2020)
Singh and Oehler, 2010 (Rousset-Jablonski, 2019)	Review (unspecified)	• General belief is that MHT should not be used, as GCTs are endocrinologically and hormonally dependent	• None
Biliatis at al., 2012 (Guidozzi, 2013)	Review (unspecified)	• MHT should not be used for GCT, as they are considered hormone dependent tumours	• None
Guidozzi, 2013 (Kuhle, 2016)	Review (unspecified)	• May be prudent to avoid estrogen therapy in survivors of GCT	• None
Kuhle et al., 2016 (O’Donnell et al., 2016)	Narrative Review	• No data on the safety of MHT after GCT, but MHT is generally avoided as GCTs are hormonally active	• Singh and Oehler, 2010 (Rousset-Jablonski, 2019)
O’Donnell et al., 2016 (Del Carmen and Rice, 2017)	Review (unspecified)	• GCTs are hormonally dependent and MHT should be avoided after diagnosis	• Biliatis et al., 2012 (Guidozzi, 2013)
Del Carmen and Rice, 2017 (Ray-Coquard, 2018)	Review (unspecified)	• No studies to refute or support use of MHT • Recommend avoiding MHT after GCT are they are hormonally active and hormone dependent	• Singh and Oehler, 2010 (Rousset-Jablonski, 2019)
Ray-Coquard et al., 2018 (Hickey, 2024)	European Society for Medical Oncology Guidelines	• MHT should be avoided, as GCTs are thought to be hormone dependent	• None
Rousset-Jablonski et al., 2019	French National Network Dedicated to Rare Gynaecological Cancers guidelines	• MHT indication should be discussed on a case to case basis in a multidisciplinary team meeting	• None
Deli et al., 2020 (Deli et al., 2020)	Review (unspecified)	• No direct evidence to prove or disprove long-term negative effect of MHT on GCT survivors • May be safer to not initiate MHT due to the endocrinologically active character of these tumours	• Singh and Oehler, 2010 (Rousset-Jablonski, 2019) • Kuhle et al., 2016 (O’Donnell et al., 2016) • Del Carmen et al., 2017 (Ray-Coquard, 2018)
Rees et al, 2020 (Rees, 2020)	European Menopause and Andropause Society and International Gynecologic Cancer Society position statement	• Generally believed that estrogens should not be used, as GCTs are hormone dependent • No study has demonstrated a deleterious effect of MHT	• None
Crean-Tate et al., 2020 (Crean-Tate et al., 2020)	Expert Review	• GCTs are hormonally active and patients are generally advised to avoid systemic MHT	• Deli et al., 2020 (Deli et al., 2020)
Cagnacci et al., 2024 (Cagnacci, 2025)	Italian Group of Study for Management of the Menopause Consensus Opinion	• MHT is allowed after non-epithelial ovarian tumors with the exception of GCT	• None
Moss et al., 2024 (Boyce, 2009)	British Gynaecological Cancer Society Practice Recommendations	• No direct evidence to support or refute the long-term negative effect of MHT on granulosa cell tumour survivors, but considering the endocrinologically active character of these tumours, it may be safer not to initiate MHT in those with advanced disease	• Deli et al., 2020 (Deli et al., 2020)
Hickey et al., 2024^61^	Review	• European guidelines suggest avoiding MHT, might be oestrogen sensitive	• Rees et al., (2020) (Rees, 2020)
Taylor et al., 2024 (Moss, 2024)	British Gynaecological Cancer Society and British Menopause Society guidelines	• Limited evidence does not demonstrate harm with MHT following treatment for stage I GCT, but tumours are hormone-sensitive and women should be counselled regarding uncertainties • MHT use, especially in advanced disease, would depend on treatment regimens, individual symptoms and risk/benefit discussions	• Rees et al., (2020)()

As seen in Table 1, none of the recommendation papers cite concrete evidence to back up the MHT recommendations in GCT patients. For example, while Singh & Ohler do not cite any evidence, many of the subsequent papers cite this paper in recommending against MHT use. (Rousset-Jablonski, 2019) Two papers, by Taylor et al. and Rousset-Jablonski et al., provide more permissive guidelines, suggesting recommending MHT on a case-by-case basis, while acknowledging that safety and risk level cannot be guaranteed with MHT use. (Taylor, 2024, Moss, 2024).

Furthermore, two papers specifically warned against the use of MHT in individuals with advanced disease. (Moss, 2024, Boyce, 2009) Both papers were published in 2024 and one of the papers, by Taylor et al. suggests that “it may be safer not to initiate MHT in those with advanced disease”. (Moss, 2024).

### 6.2 Epidemiologic evidence

There is some epidemiologic evidence investigating GCT and MHT, as well as other exogenous hormones, namely, oral contraceptives, in relation to GCT. A 2009 case-control study investigating 1578 population controls, 1511 ovarian cancer controls and 72 GCT patients found that women who had used oral contraceptives (OR 0.32; 0.17, 0.63) were less likely to develop GCT, but these results were not significant when GCT patients were compared to ovarian cancer controls. (Bryk, 2021) Another study presented prognostic factors related to survival after GCT diagnosis for 240 GCT. (Bryk, 2015) Although only 25 patients had used MHT either before or after diagnosis, this factor was not significantly related to GCT specific or overall survival. (Bryk, 2015) Another population based case control study by Bryk et al. investigated the impact and MHT on GCT, using 505 GCT patients. (Khlebus, 2023) They found that the risk of GCT did not differ significantly among women who used combined MHT for 6 months to 5 years (OR 0.23, 95% CI 0.08–0.71). (Khlebus, 2023) The authors suggest that although the study investigated initial GCT development, their results indicate that there is “no definite need to limit the use of MHT” among GCT patients. (Khlebus, 2023) These studies provide some indication that MHT may be safe in GCT patients, but the data are quite limited.

## 7 GCT recurrence

Given that the primary concern with administering MHT to GCT patients is with tumor recurrence, this section will summarize evidence around GCT recurrence, and specifically any connection between the hormonal nature of GCT and recurrence.

Studies have attempted to understand GCT recurrence through investigating hormone receptors in primary and recurrent tumors. (Němejcová, 2024, Liu, 2023, Haltia, 2020, Monteiro et al., 2024, Hutton et al., 2012) A study by Khlebus et al. on the primary and recurrent GCT microenvironment suggested that there was no difference in ERα expression between primary and recurrent tumors, when looking at 24 GCT. (Hutton et al., 2012) This is in opposition to Haltia et al., who found an increased level of ERα in recurrent tumours, with a sample size of 10, and an adjusted p-value of 0.25. (Haltia, 2020) Furthermore, in the former study by Khlebus et al. three genes important in ovarian hormone signalling (*INSL3*, *CYP19A1*, and *LHCGR*) were significantly altered in recurrent tumors. (Hutton et al., 2012) These authors observed an increased level of aromatase in recurrent tumors compared to primary ones, although they caution that this could have resulted from a complex interplay between prior hormonal treatment and genotype. (Hutton et al., 2012) Another study by Liu et al., which looked at 131 GCT, suggested that ERα score, which was based on intensity of ERα nuclear expression, was an independent risk factor for both 5 and 10 year recurrence. (Liu, 2023) Similarly, Cluzet et al. found that recurrent GCTs are more likely to express both ERα and ERβ than primary GCT, when studying 46 tumors. (Monteiro et al., 2024) They also show that ERβ is predominant over ERα in recurrent tumors. Ultimately, they hypothesize that primary and recurrent GCT response to E2 would vary according to 1) the type of E2 receptor expressed and 2) the proportion of cells that are E2 responsive. Another study by Hutton et al., of 66 GCT found that low ERβ in primary tumors was associated with a higher rate of recurrence, and recurrent tumors all showed strong and diffuse expression of ERβ. (Karalok, 2016) A study by Moh et al. found no significant differences in hormone receptor trends between primary and recurrent tumours within the same patient, suggesting some degree of stability in hormone receptor expression in patients with recurrences. (Moh, 2024) .

Some studies have also investigated the role of PR in recurrence. Hutton et al. found that there was no alteration in PR expression in recurrent GCTs compared to the primary ones. (Karalok, 2016) The previously described study by Puechl et al. used immunohistochemical staining and created a composite score based on percentage of cells staining and intensity of staining for both PR and ER expression in 149 GCT. (Puechl, 2019) ER expression was less common than PR expression (52% vs 80%) and PR composite scores were higher on average, meaning a larger proportion of cells were PR positive. (Puechl, 2019) ER composite score was not associated with recurrence, however, high PR composite scores were predictive of recurrence in this study. Evidence suggests that PR expression is associated with tumor aggressiveness in breast and endometrial cancers. (Puechl, 2019) However, the results are conflicting. With breast cancer, PR-B expression is increased in the cancerous state, but PR expression loss is associated with tumor aggressiveness. (Puechl, 2019) In opposition to this finding, the previously discussed study by Liu et al. did not find that PR score was predictive of recurrence. (Liu, 2023) While the study by Puechl et al. suggests that PR expression may be associated with GCT recurrence, there is limited evidence available and we cannot use this information to understand the impact of a MHT progestogen on GCT recurrence.

In terms of other factors related to recurrence, International Federation of Obstetrics and Gynecology (FIGO) stage at initial diagnosis is the most important prognostic factor. (Plett, 2023, Van Meurs et al., 2014, Inzani, 2022, Lukey, 2026) Other factors which have been inconsistently linked to recurrence include menopausal status, body mass index (BMI), tumor diameter, tumor mitotic index, and adjuvant therapy treatment. (Plett, 2023, Van Meurs et al., 2014, Inzani, 2022).

## 8 Discussion

There is no concrete evidence surrounding MHT in previous GCT patients. However, there is some research pertaining to the hormone receptors and hormone signaling in these tumors, which has led to weariness around MHT use in these patients. This section will discuss the findings presented in the body of the paper in the context of other relevant research.

### 8.1 Other ovarian and endometrial cancers

While there is no concrete evidence surrounding MHT and GCT, other cancers have been studied in relation to MHT. In terms of other ovarian cancers, estrogen-only MHT appears to be safe following high grade serous carcinoma, the most common ovarian cancer. (Edey et al., 2018) With regard to endometroid ovarian cancers, which are estrogen sensitive, there is some suggestion that MHT does not have detrimental effects in early stage disease. (Rees, 2020) However, Lukey et al. found that estrogen only MHT was associated with worse survival in those with endometroid cancers. (Edey et al., 2018) With respect to clear cell and mucinous carcinomas, MHT is considered reasonable, but should be individualized. (Rees, 2020) Alternatively, for low grade serous carcinoma, MHT is not recommended, as there is evidence that these tumors respond to hormone therapies, such as aromatase inhibitors. (Rees, 2020) There is very little evidence surrounding borderline malignant ovarian tumors and MHT safety. (Rees, 2020).

Endometrial cancer is hormonally active and unopposed estrogen is a major risk factor for endometroid forms, which make up approximately 80% of tumors. (Rees, 2020) Lower risk, lower grade endometroid endometrial cancers tend to be estrogen-driven, and approximately 90% of these tumors are ER positive. (Shen et al., 2017, Kreizman-Shefer et al., 2014) In endometrial cancer, ERα has a proliferative effect and ERβ is important for morphological differentiation and functional maturation. (Van Weelden, 2023) Loss of PR expression in endometrial cancer is associated with tumor aggressiveness. (Kreizman-Shefer et al., 2014) PR positive tumors have been shown to respond well to progestin therapies whereas PR negative tumors appear to respond at much lower rates (55.4% vs. 12.2%, respectively). (Mirza, 2020) In people who have had endometrial cancers that are not advanced or high risk, MHT is considered safe, despite strong ER and PR expression. (Rees, 2020, Kreizman-Shefer et al., 2014) In those with advanced stage or high risk tumors, caution is advised, and there is not sufficient evidence to draw conclusions. (Rees, 2020, Shen et al., 2017).

### 8.2 Aromatase inhibitors

In the PARAGON study the reported response rates of GCT to anastrozole were much lower than previously thought. (Banerjee, 2021) More modest reports may be a result of previous studies with good response rates investigating heavily pre-treated patients, skewing results. (Van Meurs et al., 2014, Banerjee, 2021) Furthermore, it is possible that E2 only impacts GCT when certain estrogen receptors are present. For example, Haltia et al. did not observe any changes in cell viability after letrozole treatment, even though doses were sufficient to suppress E2 production in AGCT cells. (Haltia, 2020) Their results also indicate that at physiologic doses, E2 does not impact granulosa tumours or increase cell growth. (Haltia, 2020) They recommend that because of this, MHT can be considered safe, as the levels introduced would not reach these concentrations. (Haltia, 2020) However, it is once again important to consider that MHT could have a different impact on different tumors, given that there is much variability in the receptors present. (Haltia, 2020, Monteiro et al., 2024) Additionally, Cluzet et al. found that E2 supported tumour growth, only when both ERα and ERβ were present. (Monteiro et al., 2024) Given that many tumours do not express both ERα and ERβ, E2 may not impact these tumors, and therefore aromatase inhibitors would not be beneficial. (Monteiro et al., 2024) Despite recent modest results, letrozole has shown efficacy in estrogen-receptor positive endometrial cancer when paired with cyclin dependant kinase 4/6. (Ottenbourgs, 2024) A phase II, multicentre, open label study of this combination of drugs in confirmed estrogen receptor positive GCT patients is ongoing. (Biliatis, 2012).

### 8.3 Variability in receptors

A recurring theme throughout our research is the importance of the receptors present on a tumor and the level at which they are present in determining the impact of E2 or progesterone on a tumor. In the “Estrogen Signalling in GCT” section, evidence that in the absence of both ERβ and ERα, E2 can have an anti-proliferative effect through GPER is presented. (François, 2015, Monteiro et al., 2024) Furthermore, as mentioned above, Cluzet et al. found that both ERβ and ERα must be present for E2 to exert a proliferative effect. (Monteiro et al., 2024) Combined with the findings that hormonal treatments may work better when certain hormone receptors are present, as well as the variability between GCT in the receptors present, there may be evidence to suggest that MHT use would depend on hormone receptor characterization. (Němejcová, 2024, Moh, 2024, Haltia, 2020, Ottenbourgs, 2024, Biliatis, 2012) However, this conclusion would require further evidence to support the safety of MHT use.

### 8.4 Future directions

To better understand the impact of MHT in GCT patients, there is great potential in determining exact hormone receptor status of different GCT. This status could vary between patients and tumor stage, meaning that different tumors could respond differently to exogenous hormones. (Chauvin et al., 2022) It would also be useful to understand the different responses of patients to aromatase inhibitors in the context of specific hormone receptor profiles. (Monteiro et al., 2024, Biliatis, 2012) This would allow a better understanding of the impact of E2 on different GCT. An important aspect of any future work is the use of antibodies validated for detection of hormone receptors within granulosa cells, as well as the use of non-antibody techniques to support protein detection (i.e. RNA-seq). (Birgersson, 2023, Ciucci, 2018) As well, the roles of ERβ splice variants in GCT remains poorly understood. (Birgersson, 2023) Much of the previous work on GCT used nonspecific antibodies, so the fraction of GCT that express ERs and PRs, and at what level has not been fully established. (Birgersson, 2023) Several studies have been performed in GCT cell lines that have later been found to not express ERβ. (Birgersson, 2023).

Furthermore, the downstream impacts of ERα, ERβ, GPER, and PR activation by E2 and progesterone and what hormone levels are necessary to create a response via these receptors remains mostly unknown. In particular, the regulation of *FOXL2* by E2 is not well understood. (Ito, 2022) In terms of understanding the impact of MHT, it would also be important to understand if hormone levels associated with MHT use would be sufficient to meaningfully impact these receptors, especially when compared to the hormone levels produced by a GCT itself.

## 9 Conclusion

In conclusion, there is no direct evidence suggesting that MHT is safe or unsafe for GCT patients. Despite this lack of evidence, almost all current recommendations warn against MHT use in these patients. The health and quality of life benefits associated with MHT use in individuals experiencing premature menopause cannot be discounted. However, it is important to recognize that there are also real potential harms associated with MHT, especially given there is some suggestion of proliferative effects through ERα and ERβ activation. (Haltia, 2020, François, 2015, Monteiro et al., 2024) The impact of this effect in the context of MHT is not understood and the impact of GCT hormone receptor profiles on the safety of MHT is also not well understood. Overall, there is a paucity of evidence surrounding MHT and GCT, and although these tumors are quite rare, there are women who could greatly benefit from MHT if it was shown to be safe.

## CRediT authorship contribution statement

**Abigail Shore:** Writing – review & editing, Writing – original draft, Data curation, Conceptualization. **Lanlan Fang:** Writing – review & editing, Data curation. **Shelley Hewins:** Writing – review & editing. **David G. Huntsman:** Writing – review & editing, Conceptualization. **Lesa M. Dawson:** Writing – review & editing, Supervision, Conceptualization.

## Declaration of Competing Interest

The authors declare that they have no known competing financial interests or personal relationships that could have appeared to influence the work reported in this paper.
